# *Legionella pneumophila* Serogroup 1 in the Water Facilities of a Tertiary Healthcare Center, India

**DOI:** 10.3201/eid2311.171071

**Published:** 2017-11

**Authors:** Rama Chaudhry, K. Sreenath, Valavane Arvind, E.V. Vinayaraj, Sagar Tanu

**Affiliations:** All India Institute of Medical Sciences, New Delhi, India

**Keywords:** Legionella pneumophila, water facilities, Legionnaires’ disease, legionellosis, India, bacteria, bacterial infections

## Abstract

Proactive environmental surveillance for *Legionella pneumophila* in hospitals that treat immunocompromised patients is a useful strategy for preventing nosocomial Legionnaires’ disease. We report the presence of *L. pneumophila* serogroup 1 in 15.2% of the water systems of our tertiary healthcare center, which should prompt health officials to formulate mitigation policies.

*Legionella pneumophila*, the causative agent of Legionnaires’ disease (LD), is a bacterium omnipresent in aquatic environments and increasingly recognized as a major cause of community- and hospital-acquired pneumonia. *L. pneumophila* serogroup 1 (*Lp*1), the dominant serogroup, accounts for ≈84% of human infections worldwide (*1*,*2*). Hospital-acquired LD has been reported globally, and routine use of environmental cultures is recommended as a useful strategy to prevent infections (*3*). Although proactive environmental surveillance of *Legionella* and regular treatment of cooling tower installations are recommended in many countries, these practices are not routine in India, and limited studies have been conducted in this country for monitoring *Legionella* contamination in hospital water systems (*4*). We conducted a study to detect *L. pneumophila* and to identify *Lp*1 in the water systems of a tertiary healthcare center in northern India that has organ transplantation and cancer treatment facilities.

We collected 79 water samples (41 potable, 38 nonpotable) from the hospital and general areas of the healthcare center during an 18-month period (May 2015–October 2016). Of 79 samples, 27 were collected from patient areas (wards, intensive care units, outpatient departments, emergency units, and procedure rooms); 14 from residential areas; 15 from cooling towers; and 23 from other buildings (e.g., laboratory divisions, teaching departments, library, and recreational zones). We followed guidelines issued by the US Centers for Disease Control and Prevention regarding isolation of *Legionella* (*5*). In brief, we concentrated 500 mL of water samples and decontaminated 1 part by using heat treatment (in water bath at 50°C for 30 min) and 1 part by acid (in equal volume of HCl-KCl acid buffer [pH 2.2]). We then inoculated 0.1-mL samples onto buffered charcoal yeast extract agar (Becton Dickinson, Sparks, MD, USA) supplemented with glycine, vancomycin, polymyxin B, and cycloheximide (Oxoid, Basingstoke, UK). We presumptively identified colonies growing only on buffered charcoal yeast extract but not on blood agar as *Legionella* species and confirmed the presence of *L. pneumophila* by amplification of a 375-bp region of the *mip* gene using previously published primers (*6*). We identified *Lp*1 by using a real-time PCR (rPCR) assay targeting the *wzm* gene (*7*). We used genomic DNA isolated from *L. pneumophila* strain Philadelphia (ATCC 33152) for standardization of PCR and rPCR and *L. pneumophila* strain Knoxville (ATCC 33153) for standardization of culture.

We identified *Legionella* spp. in 21 (26.6%) of 79 water samples (10 potable and 11 nonpotable) by culture. We obtained a collection of 28 isolates from the 79 samples and identified all of them as *L. pneumophila* by PCR. Among these 28 isolates, 18 (64.3%) tested positive for *Lp*1 by rPCR, indicating the presence of this pathogenic serogroup in 12 (15.2%) of the 79 water samples (5 potable and 7 nonpotable).

We repeatedly isolated *L. pneumophila* (˃4 times) from 2 high-risk sites: a drinking water unit and a cooling tower situated inside the hospital campus. Four water samples collected from patient areas tested positive for *L. pneumophila*, posing a risk for nosocomial infection. We isolated *L. pneumophila* from water bodies with temperatures ranging from 12°C to 57°C but most frequently (11 times) from those with temperatures of 25°C–50°C. We summarized the isolation of *L. pneumophila* with respect to type of water sample, sampling site, and temperature (Table).

**Table:** *Legionella pneumophila* isolated from 79 water samples collected at a tertiary healthcare center, by type of water, sampling site, and temperature, India, May 2015–October 2016

Characteristic	Samples positive for *L. pneumophila*, n = 21	Samples positive for *L. pneumophila* serogroup 1, n = 12
Type of water		
Potable	10	5
Nonpotable	11	7
Sampling site		
Patient area	4	3
Residential area	6	1
Cooling tower	5	3
Other building	6	5
Temperature, °C		
<25	9	4
25–50	11	7
>50	1	1

We demonstrated the presence of *Lp*1 in the hospital water systems, indicating that LD might be a common cause of pneumonia in this setting. Previously published reports have documented infections attributable to *L. pneumophila* in patients with community-acquired pneumonia; however, the prevalence of nosocomial legionellosis in India remains unknown (*4*,*8*,*9*).

On the basis of our findings, we initiated infection-control measures and created awareness to formulate *Legionella* risk management in this hospital. We ensured that physicians were cognizant of possible *Legionella* colonization in the hospital water supply and advised them to recommend *Legionella* diagnostic testing for patients with suspected nosocomial pneumonia. Underdiagnosed nosocomial legionellosis might become evident as the awareness of clinicians increases and as specialized laboratory testing for the pathogen becomes easily available.

Eradication of *Legionella* from aquatic bodies is a herculean task. Numerous systemic disinfection measures are available, such as super heat and flush, copper-silver ionization, chlorine dioxide, and point-of-use filters, but no method is ideal for complete eradication of the contagion (*10*). We advised the hospital’s engineers and healthcare facility managers to install point-of-use filters to the drinking water taps in areas where *Legionella* was isolated. Repeat sampling of 2 sites that had once tested positive for *Legionella* showed that they were culture-negative for the pathogen 6 six months after installation of the filters. However, to evaluate the efficacy of any disinfection method, validation and monitoring over a prolonged period is required.

Our findings might be an important sentinel of an underestimated threat and could be useful for health officials in India to set standards for LD surveillance and control in hospitals. Although we did not perform sequence-based typing in this study, examination of *Legionella* from natural sources and advances in molecular typing might help clarify the distribution and natural history of LD in this region.
